# Vibration‐Controlled Transient Elastography (VCTE)‐Based Prevalence and Assessment of Hepatic Steatosis and Fibrosis in a UK Tertiary Hospital‐Based Specialist Weight Management Service: A Real‐World Evaluation

**DOI:** 10.1002/osp4.70204

**Published:** 2026-11-05

**Authors:** Scott A. Willis, Masato Ahsan, Kiran Rathi, Ian Lawrence, Melanie J. Davies, David R. Webb, James A. King, Ehtasham Ahmad

**Affiliations:** ^1^ National Centre for Sport and Exercise Medicine School of Sport Exercise and Health Sciences Loughborough University Loughborough UK; ^2^ NIHR Leicester Biomedical Research Centre Leicester UK; ^3^ Leicester Diabetes Centre University Hospitals of Leicester NHS Trust Leicester UK; ^4^ Diabetes Research Centre University of Leicester Leicester UK

**Keywords:** fibroscan, metabolic dysfunction‐associated steatotic liver disease (MASLD), obesity, weight management services

## Abstract

**Background:**

Metabolic dysfunction‐associated steatotic liver disease is common in severe obesity; however, liver health assessment is inconsistently implemented in specialist weight management services and real‐world data are scarce. This study evaluated the prevalence and severity of vibration‐controlled transient elastography (VCTE)‐defined hepatic steatosis and fibrosis risk in adults attending a UK‐based Tier 3 specialist weight management service, and explored associations with clinical and metabolic characteristics.

**Methods:**

This retrospective cross‐sectional study included 96 adults referred to a specialist weight management service who underwent baseline VCTE (FibroScan) between June 2024 and November 2025. Controlled attenuation parameter (CAP) and liver stiffness measurement (LSM) estimated hepatic steatosis and fibrosis risk, respectively. Demographic, anthropometric, clinical, and biochemical variables were extracted from electronic records. Generalized linear models identified factors associated with CAP and LSM, with sensitivity analyses adjusting for incretin‐based therapy use.

**Results:**

Participants were predominantly female (83.3%) and White European (81.3%), with mean age 48.7 ± 12.5 years and BMI 42.0 ± 9.4 kg/m^2^. VCTE‐defined steatosis was present in 82.3% (CAP ≥ 248 dB·m^−1^), with 57.3% classified as severe (CAP ≥ 280 dB·m‐^1^). Overall, 26.1% had elevated fibrosis risk (LSM ≥ 8 kPa), including 6.3% classified as high risk (LSM > 12 kPa). In multivariable analyses, higher BMI, HbA1c and ALT were independently associated with greater CAP, whereas BMI and HbA1c were independently associated with higher LSM. Further adjustment for incretin‐based pharmacotherapy use did not alter these relationships.

**Conclusions:**

Hepatic steatosis and elevated fibrosis risk are highly prevalent in adults attending specialist weight management services. Adiposity and glycemic burden appear to be key determinants, supporting the integration of non‐invasive liver assessment into specialist obesity care pathways.

## 1 Introduction

Metabolic dysfunction‐associated steatotic liver disease (MASLD), previously termed non‐alcoholic fatty liver disease (NAFLD), is one of the most common chronic liver diseases worldwide, affecting approximately 30%–38% of adults [1, 2]. This revised nomenclature reflects the central role of metabolic dysfunction in disease pathogenesis rather than the absence of alcohol consumption as the defining feature [3]. MASLD encompasses a spectrum of liver pathology ranging from simple steatosis to metabolic dysfunction‐associated steatohepatitis (MASH), progressive fibrosis, cirrhosis, and hepatocellular carcinoma [4].

Obesity is one of the strongest drivers of MASLD onset and progression [5]. This burden is particularly marked in people with severe obesity (body mass index [BMI] ≥ 40 kg/m^2^), in whom hepatic steatosis may affect up to 90% of individuals, and the risk of MASH progression increases in parallel with rising BMI [6, 7]. MASLD commonly coexists with other obesity‐related complications such as type 2 diabetes (T2D), hypertension, obstructive sleep apnea (OSA), and chronic kidney disease, reflecting a broader phenotype of metabolic dysfunction [8, 9, 10, 11]. These metabolic conditions frequently coexist in those living with obesity and contribute to an increased risk of cardiovascular disease and premature mortality. T2D, particularly when associated with sub‐optimal glycemic control, is recognised as a risk factor for progressive liver fibrosis [12]. Identification of liver disease in people living with severe obesity is therefore important for both liver risk stratification and integrated obesity care.

Current National Institute for Health and Care Excellence (NICE) guidance recommends that individuals with risk factors for MASLD should undergo non‐invasive assessment for advanced liver fibrosis [13]. Vibration‐controlled transient elastography (VCTE; FibroScan) provides validated non‐invasive measurements of liver stiffness (LSM), as a marker of fibrosis risk, and controlled attenuation parameter (CAP), as a marker of hepatic steatosis [14, 15]. VCTE therefore offers a practical non‐invasive tool for liver risk stratification within obesity services, particularly when standard liver biochemistry including alanine aminotransferase (ALT) and aspartate aminotransferase (AST) may underestimate clinically significant liver disease [16], and liver biopsy is not often feasible for routine assessment. Implementation of VCTE within routine clinical practice, especially in obesity pathways, could facilitate earlier identification of hepatic steatosis and advanced fibrosis, enabling timely specialist referral and targeted intervention.

In the UK, Tier 3 specialist weight management services within the National Health Service (NHS) provide vital multidisciplinary care for adults with severe and complex obesity, including lifestyle, psychological, medical, and pharmacological interventions, as well as onward referral to Tier 4 bariatric services [17]. The introduction of highly effective incretin‐based weight‐lowering pharmacotherapies has further expanded the role of specialist weight management services, increasing the importance of identifying obesity‐related complications, including MASLD, that may benefit from treatment and longitudinal monitoring. These services therefore care for a population with a heavy burden of MASLD and associated liver fibrosis. Despite this risk profile and the emerging role of non‐invasive tests in MASLD assessment, routine assessment of liver health is not consistently implemented across clinical care settings including specialist weight management pathways [18, 19], and real‐world data describing the burden of hepatic steatosis and fibrosis risk in Tier 3 weight management populations are particularly sparse.

This real‐world cross‐sectional study evaluated adult attending a UK tertiary hospital‐based Tier 3 specialist weight management service who underwent VCTE assessment as part of routine clinical care. This study aimed to determine the burden of VCTE‐defined hepatic steatosis and advanced fibrosis risk, and to identify the demographic, metabolic, biochemical and socioeconomic factors associated with CAP and LSM.

## 2 Materials and Methods

### 2.1 Study Design and Population

This service evaluation presents a cross‐sectional analysis of baseline data from adults attending a UK Tier 3 specialist weight management service between June 2024 and November 2025. The main referral criteria to the service from primary care included either baseline BMI of ≥ 40 kg/m^2^ or ≥ 35 kg/m^2^ with weight‐related co‐morbidities including hypertension, hyperlipidemia, OSA, T2D and cardiovascular diseases. VCTE assessment was incorporated into routine clinical evaluation in all patients attending the service during this period as part of their baseline metabolic assessment, irrespective of current or prior incretin‐based pharmacotherapy use. Verbal informed consent was sought from all participants undergoing FibroScan.

### 2.2 Data Collection

Data were extracted from clinical letters, electronic health records, and primary care records, then pseudonymized and collated into a secure online database. Demographic variables included age, sex, and ethnicity. Socioeconomic deprivation was assessed using the Index of Multiple Deprivation (IMD) [20], which is the official measure of relative deprivation in England, derived from patient postcodes. Anthropometric measures included weight and BMI, recorded on the day of VCTE assessment. Lifestyle factors included alcohol intake and smoking status, with the latter categorized as current, former, or never smoker. Alcohol consumption of less than 14 units per week was classified as within the recommended limits. Recorded comorbidities included T2D, OSA, hypertension and, hyperlipidemia. Biochemical variables included glycated hemoglobin (HbA1c), platelet count, and alanine aminotransferase (ALT), where available within 3 months of VCTE assessment. Current use of incretin‐based pharmacotherapy was recorded as a baseline treatment variable. This included treatment with a glucagon‐like peptide‐1 receptor agonist (GLP‐1RA) (semaglutide weekly injection) or dual glucose‐dependent insulinotropic polypeptide/GLP‐1RA (tirzepatide weekly injection) at the time of VCTE assessment.

### 2.3 Vibration‐Controlled Transient Elastography (FibroScan)

All VCTE assessments were performed using a FibroScan Model 430 mini + device (SN F93528; Echosens, Paris, France) by a trained nurse blinded to the clinical and laboratory data. To account for varying body habitus, either a Medium (M) or Large (XL) probe was utilized as clinically indicated.

LSM was recorded in kilopascals (kPa) as a non‐invasive marker of fibrosis risk. Fibrosis risk categories were defined using the following thresholds: low risk, < 8 kPa; indeterminate risk, 8–12 kPa; and high risk, > 12 kPa [21]. CAP was recorded in dB·m^−1^ as a non‐invasive marker of hepatic steatosis. Steatosis grades were defined as: S0 (no steatosis), < 248 dB·m^−1^; S1 (mild), 248–267 dB·m^−1^; S2 (moderate), 268–279 dB·m^−1^; and S3 (severe), ≥ 280 dB·m^−1^ [22].

### 2.4 Data Quality and Reliability

To ensure the technical validity of the FibroScan results, the interquartile range (IQR) and IQR‐to‐median ratio for LSM measurements were recorded for each session. Results were considered highly reliable when the IQR‐to‐median ratio was ≤ 0.30.

### 2.5 Statistical Analysis

All analyses were conducted using IBM SPSS Statistics (Version 31; IBM Corp., Armonk, NY, USA). Kolmogorov–Smirnov tests and visual inspection of histograms were used to assess the normality of continuous variables. Normally distributed data are presented as means ± standard deviations (SD), and non‐normally distributed data as median (interquartile range [IQR]). Categorical variables are presented as number [column percentage].

CAP and LSM were analyzed as continuous outcomes. Univariable associations between clinical variables and CAP were assessed using generalized linear models assuming a normal distribution with an identity link function. Due to positive skewness of LSM, univariable associations were assessed using generalized linear models assuming a gamma distribution with an identity link function.

Multivariable models were subsequently constructed separately for CAP and LSM to identify independent clinical predictors of hepatic steatosis and fibrosis risk. Covariates were selected a priori based on clinical relevance and established associations with MASLD, and included age, sex, ethnicity, IMD, alcohol use, BMI, HbA1c, and ALT. Distributional assumptions were consistent with the corresponding univariable models.

Given the high prevalence of incretin‐based pharmacotherapy use within the Tier 3 weight management cohort, sensitivity analyses were performed to evaluate whether the observed associations were independent of current incretin exposure. Multivariable models were repeated with additional adjustment for incretin‐based pharmacotherapy use (categorized as any use vs. none), and effect estimates were compared to the primary models to assess robustness of associations. Regression coefficients (*β*), 95% confidence intervals (CI), and *p*‐values are reported, with a two‐sided *p*‐value < 0.05 considered statistically significant.

## 3 Results

### 3.1 Cohort Characteristics

A total of 482 patients attended various arms of the Tier 3 service; however, the current cohort only included 96 adults who underwent baseline VCTE assessment as part of routine clinical care when the FibroScan facility was made available during the latter part of the study for those attending face to face clinical consultations only. The full Fibroscan cohort characteristics are summarized in Table 1. The cohort was predominantly female (83.3%) and White European (81.3%), with a mean age of 48.7 ± 12.5 years and mean BMI of 42.0 ± 9.4 kg/m^2^·. Cardiometabolic comorbidities were common, including T2D (20.8%), hypertension (45.8%), hyperlipidemia (39.6%), and OSA (29.2%). Incretin‐based pharmacotherapy was used by 47.9% of participants. At the time of VCTE assessment, the participants receiving semaglutide had a median dose of 2.4 mg weekly and mean treatment duration of 47.3 weeks, while those receiving tirzepatide therapy had a median dose of 10 mg weekly and a mean treatment duration of 35.4 weeks.

**TABLE 1:** Cohort characteristics.

Characteristic	All (*n* = 96)
Demographics	
Age (years)	48.7 ± 12.5
Sex	
Female [%]	80 [83.3]
Male [%]	16 [16.7]
Ethnicity	
White European [%]	78 [81.3]
South Asian [%]	15 [15.6]
Black [%]	2 [2.1]
Other [%]	1 [1.0]
IMD (decile)	6 (5)
Smoking status	
Non‐smoker [%]	79 [82.3]
Former smoker [%]	14 [14.6]
Current smoker [%]	3 [3.1]
Alcohol use [%]	
No [%]	91 [94.8]
Yes [%]	5 [5.2]
Anthropometric and biochemical profile	
Weight (kg)	116.7 (36.5)
BMI (kg/m^2^)	42.0 (9.4)
HbA1c (mmol/mol)	38.0 (7.0)
ALT (U/L)	22.0 (14.0)
Platelet count (× 10^9^·L^−1^) (n = 89)	286 (74)
Liver VCTE assessment	
CAP (dB/m)	293 ± 55
Steatosis grade	
S0 (< 248 dB·m^−1^) [%]	17 [17.7]
S1 (248‐267 dB·m^−1^) [%]	15 [15.6]
S2 (268‐279 dB·m^−1^) [%]	9 [9.4]
S3 (≥ 280 dB·m^−1^) [%]	55 [57.3]
LSM (kPa)	6.1 (3.4)
Advanced fibrosis risk	
Low (< 8 kPa) [%]	71 [74.0]
Indeterminate (8‐12 kPa) [%]	19 [19.8]
High (> 12 kPa) [%]	6 [6.3]
Medical history	
Type 2 diabetes	
No [%]	76 [79.2]
Yes [%]	20 [20.8]
Hypertension	
No [%]	52 [54.2]
Yes [%]	44 [45.8]
Hyperlipidemia	
No [%]	58 [60.4]
Yes [%]	38 [39.6]
Obstructive sleep apnea	
No [%]	68 [70.8]
Yes [%]	28 [29.2]
Incretin‐based therapy use	
None [%]	50 [52.1]
Semaglutide [%]	20 [20.8]
Tirzepatide [%]	26 [27.1]

*Note:* Data are presented as mean ± standard deviation, median (interquartile range), or number [column percentage] where appropriate.

Abbreviations: ALT, alanine aminotransferase; ASCVD, atherosclerotic cardiovascular disease; BMI, body mass index; CAP, controlled attenuation parameter; CKD, chronic kidney disease; HbA1c, glycated hemoglobin; IMD, index of multiple deprivation; LSM, liver stiffness measurement; VCTE, vibration‐controlled transient elastography.

Mean CAP was 293 ± 55 dB·m^−1^, while median LSM was 6.1 (IQR 3.4) kPa. Cohort characteristics stratified by steatosis grade, fibrosis risk category, and incretin‐based pharmacotherapy use are presented in Tables [Link], [Link], [Link].

### 3.2 Prevalence of Hepatic Steatosis and Fibrosis

Evidence of hepatic steatosis (S1‐S3) was present in 82.3% of participants, with 57.3% classified as severe (S3; CAP ≥ 280 dB·m^−1^) (Table 1). Overall, 26.1% of participants had indeterminate or high risk of advanced fibrosis (LSM ≥ 8 kPa), including 6.3% classified as high risk (> 12 kPa) (Table 1). Increasing steatosis grade was associated with higher median LSM values, with 41.8% of individuals with severe steatosis (S3) classified as having indeterminate or high fibrosis risk.

### 3.3 Univariable Associations With CAP and LSM

Univariable analyses are presented in Tables 2 and 3 for CAP and LSM, respectively. Body weight, BMI, and LSM were positively associated with CAP (all *p* < 0.001). Furthermore, HbA1c and ALT showed trends toward association with CAP (both *p* ≤ 0.067). For LSM, body weight, BMI, HbA1c, and CAP were positively associated (all *p* ≤ 0.012), while ALT demonstrated a borderline association (*p* = 0.061). Age, sex, ethnicity, IMD, alcohol use, and comorbidities were not associated with CAP or LSM in univariable analyses.

**TABLE 2:** Univariable regression analysis of factors associated with VCTE‐assessed hepatic steatosis (CAP).

Variable	ß‐coefficient	95% CI	*p*‐value
Age (per year)	−0.18	−1.06 to 0.70	0.691
Female sex	−13.06	−42.21 to 16.09	0.380
Non‐white ethnicity	2.39	−25.55 to 30.33	0.867
IMD (per decile)	2.07	−1.60 to 5.76	0.269
Ever smoking	16.37	−12.01 to 44.76	0.258
Alcohol use	6.00	−43.08 to 55.07	0.811
Weight (per kg)	0.94	0.60 to 1.28	< 0.001
BMI (per kg/m^2^)	3.09	1.85 to 4.33	< 0.001
HbA1c (per mmol/mol)	1.24	−0.09 to 2.57	0.067
ALT (per U/L)	0.69	−0.01 to 1.39	0.054
Platelet count (per × 10^9^·L^−1^)^a^	−0.05	−0.24 to 0.14	0.589
LSM (per kPa)	8.95	5.81 to 12.09	< 0.001
Type 2 diabetes	16.21	−10.45 to 42.88	0.233
Hypertension	1.80	−20.09 to 23.69	0.872
Hyperlipidemia	−8.42	−30.66 to 13.82	0.458
Obstructive sleep apnea	15.32	−8.48 to 39.12	0.207
Incretin‐based therapy use	−15.83	−37.34 to 5.77	0.151

*Note:* Data are presented as ß‐coefficients, 95% CIs, and *p*‐values from univariable generalized linear models assuming a normal distribution and identity link function. ß‐coefficients represent the mean change in CAP (dB·m^−1^) per unit increase in the predictor variable. Binary variables are compared against the reference category (male sex, White ethnicity, never smoker, no alcohol use, absence of comorbidity, and no incretin‐based therapy use).

Abbreviations: ALT, alanine aminotransferase; BMI, body mass index; CAP, controlled attenuation parameter; CI, confidence interval; HbA1c, glycated hemoglobin; IMD, index of multiple deprivation; LSM, liver stiffness measurement; VCTE, vibration‐controlled transient elastography.

^a^ indicates *n* = 89 for platelet count.

**TABLE 3:** Univariable regression analysis of factors associated with VCTE‐assessed hepatic fibrosis (LSM).

Variable	ß‐coefficient	95% CI	*p*‐value
Age (per year)	−0.00	−0.05 to 0.04	0.887
Female sex	−0.45	−2.07 to 1.17	0.585
Non‐white ethnicity	0.39	−1.14 to 1.93	0.614
IMD (per decile)	−0.03	−0.22 to 0.16	0.764
Ever smoking	0.35	−1.21 to 1.92	0.658
Alcohol use	1.66	−1.50 to 4.82	0.304
Weight (per kg)	0.03	0.01 to 0.05	0.002
BMI (per kg/m^2^)	0.15	0.07 to 0.23	< 0.001
HbA1c (per mmol/mol)	0.12	0.03 to 0.21	0.012
ALT (per U/L)	0.05	−0.00 to 0.10	0.061
Platelet count (per × 10^9^ ·L^−1^)^a^	0.00	−0.01 to 0.01	0.950
CAP (per dB·m^−1^)	0.03	0.02 to 0.04	< 0.001
Type 2 diabetes	1.32	−0.25 to 2.88	0.100
Hypertension	0.73	−0.44 to 1.89	0.222
Hyperlipidemia	−0.50	−1.66 to 0.66	0.401
Obstructive sleep apnea	0.58	−0.73 to 1.90	0.383
Incretin‐based therapy	−0.61	−1.75 to 0.54	0.299

*Note:* Data are presented as ß‐coefficients, 95% CIs, and *p*‐values from univariable generalized linear models assuming a gamma distribution and identity link function. ß‐coefficients represent the mean change in LSM (kPa) per unit increase in the predictor variable. Binary variables are compared against the reference category (male sex, White ethnicity, never smoker, no alcohol use, absence of comorbidity, and no incretin‐based therapy use).

Abbreviations: ALT, alanine aminotransferase; BMI, body mass index; CAP, controlled attenuation parameter; CI, confidence interval; HbA1c, glycated hemoglobin; IMD, index of multiple deprivation; LSM, liver stiffness measurement; VCTE, vibration‐controlled transient elastography.

^a^ indicates *n* = 89 for platelet count.

### 3.4 Multivariable Associations With CAP and LSM

Multivariable analyses for CAP and LSM are presented in Table 4. In adjusted models, higher BMI and HbA1c were independently associated with greater CAP. Specifically, each 1 kg/m^2^ increase in BMI was associated with a 3.22 dB·m^−1^ increase in CAP (95% CI: 2.05 to 4.39; *p* < 0.001), while each 1 mmol/mol increase in HbA1c was associated with a 1.35 dB·m^−1^ increase (95% CI: 0.15 to 2.55; *p* = 0.027). ALT was also independently associated with CAP (β: 0.70 per U/L; 95% CI: 0.06 to 1.33; *p* = 0.031). Age, sex, ethnicity, IMD, and alcohol use were not independently associated.

**TABLE 4:** Multivariable regression analysis of factors associated with VCTE‐assessed hepatic steatosis (CAP) and fibrosis (LSM).

Variable	CAP			LSM		
	ß‐coefficient	95% CI	*p*‐value	ß‐coefficient	95% CI	*p*‐value
Age (per year)	−0.13	−0.89 to 0.63	0.741	−0.00	−0.04 to 0.03	0.830
Female sex	−1.18	−27.51 to 25.16	0.930	0.13	−1.29 to 1.56	0.853
Non‐white ethnicity	10.26	−14.74 to 35.27	0.421	0.57	−0.77 to 1.91	0.404
IMD (per decile)	2.26	−1.02 to 5.54	0.176	0.04	−0.13 to 0.20	0.658
Alcohol use	−6.40	−49.57 to 36.77	0.771	0.75	−1.80 to 3.29	0.565
BMI (per kg/m^2^)	3.22	2.05 to 4.39	< 0.001	0.15	0.08 to 0.22	< 0.001
HbA1c (per mmol/mol)	1.35	0.15 to 2.55	0.027	0.12	0.04 to 0.20	0.003
ALT (per U/L)	0.70	0.06 to 1.33	0.031	0.03	−0.01 to 0.07	0.091

*Note:* Data are presented as ß‐coefficients, 95% CIs, and *p*‐values from multivariable generalized linear models assuming a normal (CAP) or gamma (LSM) distribution with identity link function. ß‐coefficients represent the mean change in CAP (dB·m^−1^) or LSM (kPa) per unit increase in the predictor variable. Binary variables are compared against the reference category (male sex, White ethnicity, no alcohol use). Model covariates included age, sex, ethnicity, IMD, alcohol use, BMI, HbA1c, and ALT. ALT, alanine aminotransferase.

Abbreviations: BMI, body mass index; CAP, controlled attenuation parameter; CI, confidence interval; HbA1c, glycated hemoglobin; IMD, index of multiple deprivation; LSM, liver stiffness measurement; VCTE, vibration‐controlled transient elastography.

For LSM, BMI and HbA1c were also independently associated with higher liver stiffness. Specifically, each 1 kg/m^2^ increase in BMI was associated with a 0.15 kPa increase in LSM (95% CI: 0.08 to 0.22; *p* < 0.001), while each 1 mmol/mol increase in HbA1c was associated with a 0.12 kPa increase (95% CI: 0.04 to 0.20, *p* = 0.003). No independent associations were observed for age, sex, ethnicity, IMD, alcohol use, or ALT.

### 3.5 Sensitivity Analysis

Additional adjustment for incretin‐based pharmacotherapy use did not materially alter the observed associations (Table S4). BMI and HbA1c remained independently associated with both CAP and LSM (all *p* ≤ 0.027), while incretin‐based therapy use was not independently associated with either outcome.

## 4 Discussion

This real‐world cross‐sectional analysis of adults attending a UK tertiary hospital‐based Tier 3 specialist weight management service demonstrates a great burden of MASLD, with over 80% exhibiting VCTE‐defined hepatic steatosis and more than half meeting the criteria for severe steatosis (57.3%). Additionally, approximately one‐quarter (26.1%) of the cohort exhibited an elevated risk of advanced fibrosis, with 6.3% classified as having a high risk. Across both hepatic steatosis (CAP) and fibrosis risk (LSM), adiposity and glycemic status emerged as key independent determinants in this severe and complex obesity population. Conversely, demographic factors, socioeconomic deprivation, alcohol use, and other comorbidities were not independently associated. Adjustment for baseline incretin‐based therapy use did not alter these associations.

The prevalence of hepatic steatosis in this cohort (82.3%) is consistent with previous studies reporting hepatic steatosis in up to 80%–90% of individuals with severe obesity [6, 7]. The study findings extend this literature by providing real‐world data from a UK Tier 3 specialist weight management service, where patients typically have severe obesity, complex multimorbidity, and high metabolic risk, but structured liver assessment is not consistently embedded. The high proportion of patients with severe steatosis (57.3%) further highlights the extent of liver fat accumulation in this clinical population and supports MASLD as a common obesity‐related complication within specialist weight management pathways.

The strong independent association between BMI and CAP supports established evidence that adiposity is a primary driver of hepatic fat accumulation [5, 23]. In severe obesity, adipose tissue dysfunction, insulin resistance, and ectopic lipid deposition promote hepatic triglyceride accumulation and MASLD progression. The independent association between HbA1c and CAP further supports the role of glycemic dysregulation in hepatic steatosis, consistent with the literature linking insulin resistance, hyperglycaemia, and increased hepatic *de novo* lipogenesis with intrahepatic lipid deposition [24, 25, 26]. Notably, this association was observed using HbA1c as a continuous measure, suggesting that liver fat may vary across the spectrum of glycemic status rather than only according to T2D diagnosis.

These findings are consistent with previous VCTE‐based studies in populations with obesity and metabolic dysfunction. In a general internal medicine clinic cohort, CAP was positively associated with body weight, BMI, ALT and HbA1c in univariable analyses, with BMI remaining an independent predictor of hepatic steatosis in multivariable modeling [27]. Similar associations between BMI, glycemic dysfunction, and hepatic steatosis have been reported in larger cross‐sectional cohorts of individuals living with obesity and metabolic derangements [26, 28]. Overall, these findings reinforce the central role of adiposity and glycemic status in hepatic fat accumulation.

In general population cohorts, the prevalence of advanced fibrosis is typically low, at around 3%–5% [29, 30]. In contrast, 26.1% of individuals in our Tier 3 cohort had elevated fibrosis risk, including 6.3% classified as high risk. This higher prevalence likely reflects the enriched metabolic risk profile of patients referred to specialist weight management services who commonly have severe obesity and cardiometabolic multimorbidity. Notably, 41.8% of individuals with severe steatosis also had indeterminate or high fibrosis risk (LSM ≥ 8 kPa), suggesting that a clinically relevant subgroup within obesity services may warrant further liver assessment or hepatology referral.

The independent associations between BMI, HbA1c, and LSM observed in this study are consistent with evidence that fibrosis risk increases with worsening metabolic dysfunction [31, 32]. In patients with MASLD, higher BMI has been associated with higher LSM and greater odds of more advanced fibrosis [33], with similar associations reported in larger real‐world prospective studies [34, 35]. Glycemic status is also a major contributor, with T2D, longer diabetes duration, and sub‐optimal glycemic control associated with increased fibrosis risk in MASLD [28]. Meta‐analyses further report advanced fibrosis in 17%–20% of individuals with T2D [36, 37]. Together, these findings additionally support adiposity and glycemic dysregulation as central drivers of fibrosis risk and MASLD progression in severe and complex obesity. This is consistent with evidence that substantial weight loss through bariatric surgery [38] or pharmacotherapy can improve hepatic steatosis and fibrosis‐related outcomes [39, 40].

Notably, ALT was independently associated with CAP but not LSM in adjusted analyses, suggesting that ALT may better reflect hepatic steatosis or steatosis‐related hepatocellular injury than fibrosis risk in this cohort. This is clinically important, as routine liver enzymes may not reliably identify patients at risk of advanced fibrosis, particularly in severe obesity where liver disease may be present despite normal or only mildly abnormal liver biochemistry. These findings support the use of other non‐invasive assessment tools, such as VCTE, rather than reliance on liver function tests alone within specialist weight management pathways.

The inclusion of incretin‐based therapy use in sensitivity analyses did not materially alter the observed associations between BMI, HbA1c and VCTE‐derived measures of steatosis and fibrosis, supporting the robustness of the main study findings. Although incretin‐based therapy use was not found to be independently associated with CAP or LSM after multivariable adjustment, this finding should be interpreted cautiously. Participants receiving semaglutide and tirzepatide had received clinically meaningful treatment exposure prior to FibroScan assessment. At the time of VCTE assessment, participants receiving semaglutide had a median dose of 2.4 mg weekly and mean treatment duration of 47.3 weeks, whereas those receiving tirzepatide had a median dose of 10 mg weekly and mean treatment duration of 35.4 weeks. Patients receiving incretin‐based therapies had lower baseline BMI and body weight compared with untreated individuals, suggesting potential confounding effects of indication and baseline metabolic differences. Therefore, the absence of an observed association should not be interpreted as evidence of no therapeutic effect, particularly given randomised controlled trial evidence that GLP‐1RAs and dual GIP/GLP‐1Ras inducing > 10% weight loss from baseline can improve liver‐related outcomes in MASLD/MASH [41, 42, 43]. Such magnitudes of weight loss have historically been difficult to achieve through lifestyle intervention alone, highlighting the potential importance of modern pharmacotherapies in this setting. However, longitudinal studies with repeated VCTE assessments are required to evaluate treatment effects on liver outcomes in real‐world specialist obesity services.

By characterizing liver health in this under‐studied high‐risk clinical population, the findings from this study provide real‐world evidence to inform the integration of liver assessment and risk stratification into specialist obesity care pathways. The findings have important potential implications for clinical practice and provide clinically relevant insights into the under‐recognised burden of liver disease in both primary and secondary care specialist services. The high prevalence of VCTE‐defined hepatic steatosis and elevated fibrosis risk supports routine integration of non‐invasive liver assessment within specialist weight management pathways, and potentially within primary care and community pathways for individuals living with severe obesity. This is particularly relevant given the anticipated shift in NHS obesity management from secondary care specialist settings to primary care and community‐based services. VCTE offers a practical, non‐invasive tool for early liver risk stratification and identification of individuals requiring hepatology referral. While VCTE provides direct assessment of hepatic steatosis and fibrosis, alternative validated approaches including FIB‐4 and enhanced liver fibrosis (ELF) testing may also provide pragmatic options for wider implementation across primary care and community settings.

The consistent associations of BMI and HbA1c with both CAP and LSM reinforce the importance of weight loss and glycemic optimisation as central components of liver risk reduction. Given evidence that ≥ 10% weight loss can reverse MASH and improve fibrosis [44], effective weight‐loss pharmacotherapies may represent a key strategy for reducing liver‐related morbidity in high‐risk populations, such as those with LSM > 8 kPa. Several emerging therapies are in development specifically targeting at‐risk MASH [40, 45].

This study has several strengths, including objective VCTE‐derived measures within a UK specialist weight management setting, detailed phenotyping of a real‐world Tier three population with severe and complex obesity, and multivariable modeling of clinically relevant covariates. However, several limitations should be acknowledged. First, the cross‐sectional design precludes causal inference and assessment of temporal relationships. Second, histological confirmation was unavailable although VCTE is recognized as a validated non‐invasive tool for assessing hepatic steatosis and fibrosis risk [46, 47]. Third, this was a single‐center study with a relatively modest sample size, limiting generalizability and statistical power. Fourth, some variables of interest, such as AST levels and FIB‐4 scores, were incomplete or unavailable, which may have influenced adjusted associations. Fourth, as VCTE was introduced later during the Tier 3 service, only a subset of the overall Tier three population underwent FibroScan assessment; therefore, selection bias cannot be excluded. Finally, residual confounding cannot be excluded, particularly for incretin‐based therapy use, where baseline differences between groups may have influenced observed associations.

## 5 Conclusion

In a Tier 3 specialist weight management cohort undergoing baseline VCTE assessment, MASLD burden and elevated fibrosis risk were highly prevalent. Adiposity and glycemic burden appear to be key determinants of both hepatic steatosis and fibrosis risk in this severe and complex obesity population. These findings support the routine integration of non‐invasive liver assessment into obesity care pathways and justify longitudinal evaluation of whether modern weight‐loss therapies improve liver outcomes in real‐world settings.

## Author Contributions

E.A., D.R.W., J.A.K. and S.A.W. contributed to the conceptualization and design of the study. M.A. and K.R. were responsible for data collection. S.A.W. and J.A.K. performed the statistical analyses. S.A.W., M.A., K.R. and E.A. contributed to manuscript drafting and finalization. I.L., M.J.D., D.R.W. and J.A.K. critically reviewed and revised the manuscript for important intellectual content. All authors reviewed and approved the final version of the manuscript.

## Ethics Statement

This work was conducted as a retrospective service evaluation using routinely collected anonymized clinical data obtained as part of standard care within a Tier 3 specialist weight management service. In accordance with UK Health Research Authority guidance, NHS Research Ethics Committee approval was not required. All data were pseudonymized prior to analysis and handled in accordance with local information governance policies.

## Conflicts of Interest

The authors declare no conflicts of interest.

## Supporting information


**Table S1:** Cohort characteristics stratified by CAP‐derived steatosis grade.

**Table S2:** Cohort characteristics stratified by LSM‐derived advanced fibrosis risk category.

**Table S3:** Cohort characteristics stratified by incretin‐based therapy use.

**Table S4:** Sensitivity analysis of the multivariable models of factors associated with VCTE‐assessed hepatic steatosis (CAP) and fibrosis (LSM) with additional adjustment for incretin‐based therapy use.

## Data Availability

The data that support the findings of this study are available from the corresponding author upon reasonable request.
