# Supplementary material for: Vibration‐Controlled Transient Elastography (VCTE)‐Based Prevalence and Assessment of Hepatic Steatosis and Fibrosis in a UK Tertiary Hospital‐Based Specialist Weight Management Service: A Real‐World Evaluation
Source: Obes Sci Pract. 2026 Nov 5;12(6):e70204. doi: 10.1002/osp4.70204 (PMC13735886; doi:10.1002/osp4.70204)
Supplement: Supplementary file 1 [file OSP4-12-e70204-s001.docx]

**Supplementary Table 1.** Cohort characteristics stratified by CAP-derived steatosis grade.

| **Characteristic** | **S0 (<248 dB∙m^-1^)**  **(*n* = 17)** | **S1 (248-267 dB∙m^-1^)**  **(*n* = 15)** | **S2 (268-279 dB∙m^-1^)**  **(*n* = 9)** | **S3 (≥280 dB∙m^-1^)**  **(*n* = 55)** |
| --- | --- | --- | --- | --- |
| **Demographics** | |  |  |  |
| Age (years) | 50.8 ± 12.5 | 48.7 ± 13.4 | 46.7 ± 9.1 | 48.3 ± 12.9 |
| Sex |  |  |  |  |
| *Female [%]* | 15 (88.2) | 14 (93.3) | 8 (88.9) | 43 (78.2) |
| *Male [%]* | 2 (11.8) | 1 (6.7) | 1 (11.1) | 12 (21.8) |
| Ethnicity |  |  |  |  |
| *White European [%]* | 14 (82.4) | 12 (80.0) | 7 (77.8) | 45 (81.8) |
| *South Asian [%]* | 3 (17.6) | 3 (20.0) | 2 (22.2) | 7 (12.7) |
| *Black [%]* | 0 (0.0) | 0 (0.0) | 0 (0.0) | 2 (3.6) |
| *Other [%]* | 0 (0.0) | 0 (0.0) | 0 (0.0) | 1 (1.8) |
| IMD (decile) | 6 (5) | 7 (6) | 6 (6) | 6 (4) |
| Smoking status |  |  |  |  |
| *Non-smoker [%]* | 14 (82.4) | 14 (93.3) | 8 (88.9) | 43 (78.2) |
| *Former smoker [%]* | 3 (17.6) | 1 (6.7) | 1 (11.1) | 9 (16.4) |
| *Current smoker [%]* | 0 (0.0) | 0 (0.0) | 0 (0.0) | 3 (5.5) |
| Alcohol use [%] |  |  |  |  |
| No *[%]* | 16 (94.1) | 15 (100.0) | 9 (100.0) | 51 (92.7) |
| Yes *[%]* | 1 (5.9) | 0 (0.0) | 0 (0.0) | 4 (7.3) |
| **Anthropometric and biochemical profile** | |  |  |  |
| Weight (kg) | 98.4 (28.9) | 103.0 (29.9) | 115.4 (33.7) | 124.9 (39.2) |
| BMI (kg/m^2^) | 38.2 (7.3) | 40.2 (8.7) | 43.1 (12.3) | 44.5 (12.4) |
| HbA1c (mmol/mol) | 37.0 (6.0) | 34.0 (10.0) | 38.0 (7.0) | 38.0 (6.0) |
| ALT (U/L) | 18.0 (11.0) | 18.0 (10.0) | 24.0 (12.0) | 23.0 (17.0) |
| Platelet count (×10^9^∙L^-1^) | 286 (105) | 300 (76) | 301 (81) | 274 (68) |
| **Liver VCTE assessment** | |  |  |  |
| CAP (dB/m) | 213 ± 27 | 259 ± 6 | 274 ± 3 | 330 ± 35 |
| LSM (kPa) | 5.1 (2.9) | 4.8 (2.0) | 4.8 (2.9) | 7.3 (4.6) |
| Advanced fibrosis risk |  |  |  |  |
| *Low (<8 kPa) [%]* | 16 (94.1) | 15 (100.0) | 8 (88.9) | 32 (58.2) |
| *Indeterminate (8-12 kPa) [%]* | 1 (5.9) | 0 (0.0) | 1 (11.1) | 17 (30.9) |
| *High (>12 kPa) [%]* | 0 (0.0) | 0 (0.0) | 0 (0.0) | 6 (10.9) |
| **Medical history** | |  |  |  |
| Type 2 diabetes |  |  |  |  |
| *No [%]* | 14 (82.4) | 13 (86.7) | 7 (77.8) | 42 (76.4) |
| *Yes [%]* | 3 (17.6) | 2 (13.3) | 2 (22.2) | 13 (23.6) |
| Hypertension |  |  |  |  |
| *No [%]* | 9 (52.9) | 9 (60.0) | 3 (33.3) | 31 (56.4) |
| *Yes [%]* | 8 (47.1) | 6 (40.0) | 6 (66.7) | 24 (43.6) |
| Hyperlipidaemia |  |  |  |  |
| *No [%]* | 10 (58.8) | 10 (66.7) | 3 (33.3) | 35 (63.6) |
| *Yes [%]* | 7 (41.2) | 5 (33.3) | 6 (66.7) | 20 (36.4) |
| Obstructive sleep apnoea |  |  |  |  |
| *No [%]* | 13 (76.5) | 11 (73.3) | 7 (77.8) | 37 (67.3) |
| *Yes [%]* | 4 (23.5) | 4 (26.7) | 2 (22.2) | 18 (32.7) |
| Incretin-based therapy use |  |  |  |  |
| *None [%]* | 7 (41.2) | 5 (33.3) | 5 (55.6) | 33 (60.0) |
| *Semaglutide [%]* | 3 (17.6) | 4 (26.7) | 0 (0.0) | 13 (23.6) |
| *Tirzepatide [%]* | 7 (41.2) | 6 (40.0) | 4 (44.4) | 9 (16.4) |

Data are presented as mean ± standard deviation, median (interquartile range), or number [column percentage] where appropriate. *indicates n = 15 for S0, n = 8 for S2, and n = 51 for S3. ALT, alanine aminotransferase; ASCVD, atherosclerotic cardiovascular disease; BMI, body mass index; CAP, controlled attenuation parameter; CKD, chronic kidney disease; HbA1c, glycated haemoglobin; IMD, index of multiple deprivation; LSM, liver stiffness measurement; VCTE, vibration-controlled transient elastography.
