# Supplementary material for: Vibration‐Controlled Transient Elastography (VCTE)‐Based Prevalence and Assessment of Hepatic Steatosis and Fibrosis in a UK Tertiary Hospital‐Based Specialist Weight Management Service: A Real‐World Evaluation
Source: Obes Sci Pract. 2026 Nov 5;12(6):e70204. doi: 10.1002/osp4.70204 (PMC13735886; doi:10.1002/osp4.70204)
Supplement: Supplementary file 2 [file OSP4-12-e70204-s002.docx]

**Supplementary Table 2.** Cohort characteristics stratified by LSM-derived advanced fibrosis risk category.

| **Characteristic** | **Low (<8 kPa)**  **(*n* = 71)** | **Indeterminate (8-12 kPa)**  **(*n* = 19)** | **High (>12 kPa)**  **(*n* = 6)** |
| --- | --- | --- | --- |
| **Demographics** | |  |  |
| Age (years) | 48.7 ± 12.3 | 47.5 ± 14.4 | 52.2 ± 8.9 |
| Sex |  |  |  |
| *Female [%]* | 60 (84.5) | 15 (78.9) | 5 (83.3) |
| *Male [%]* | 11 (15.5) | 4 (21.1) | 1 (16.7) |
| Ethnicity |  |  |  |
| *White European [%]* | 60 (84.5) | 13 (68.4) | 5 (83.3) |
| *South Asian [%]* | 10 (14.1) | 5 (26.3) | 0 (0.0) |
| *Black [%]* | 1 (1.4) | 0 (0.0) | 1 (16.7) |
| *Other [%]* | 0 (0.0) | 1 (5.3) | 0 (0.0) |
| IMD (decile) | 6 (5) | 6 (5) | 5 (7) |
| Smoking status |  |  |  |
| *Non-smoker [%]* | 59 (83.1) | 15 (78.9) | 5 (83.3) |
| *Former smoker [%]* | 10 (14.1) | 3 (15.8) | 1 (16.7) |
| *Current smoker [%]* | 2 (2.8) | 1 (5.3) | 0 (0.0) |
| Alcohol use [%] |  |  |  |
| No *[%]* | 69 (97.2) | 17 (89.5) | 5 (83.3) |
| Yes *[%]* | 2 (2.8) | 2 (10.5) | 1 (16.7) |
| **Anthropometric and biochemical profile** | |  |  |
| Weight (kg) | 114.9 (30.6) | 128.0 (55.6) | 124.1 (55.9) |
| BMI (kg/m^2^) | 41.2 (8.9) | 44.5 (13.9) | 48.1 (16.8) |
| HbA1c (mmol/mol) | 37.0 (7.0) | 38.0 (8.0) | 41.5 (15.0) |
| ALT (U/L) | 20.0 (10.0) | 30.0 (25.0) | 27.5 (15.0) |
| Platelet count (×10^9^∙L^-1^) | 289 (73) | 274 (83) | 300 (94) |
| **Liver VCTE assessment** | |  |  |
| CAP (dB/m) | 281 ± 54 | 320 ± 40 | 354 ± 39 |
| Steatosis grade |  |  |  |
| *S0 (<248 dB∙m^-1^) [%]* | 16 (22.5) | 1 (5.3) | 0 (0.0) |
| *S1 (248-267 dB∙m^-1^) [%]* | 15 (21.1) | 0 (0.0) | 0 (0.0) |
| *S2 (268-279 dB∙m^-1^) [%]* | 8 (11.3) | 1 (5.3) | 0 (0.0) |
| *S3 (≥280 dB∙m^-1^) [%]* | 32 (45.1) | 17 (89.5) | 6 (100.0) |
| LSM (kPa) | 5.2 (2.1) | 9.7 (2.6) | 13.1 (2.6) |
| **Medical history** | |  |  |
| Type 2 diabetes |  |  |  |
| *No [%]* | 57 (80.3) | 15 (78.9) | 4 (66.7) |
| *Yes [%]* | 14 (19.7) | 4 (21.1) | 2 (33.3) |
| Hypertension |  |  |  |
| *No [%]* | 39 (54.9) | 12 (63.2) | 1 (16.7) |
| *Yes [%]* | 32 (45.1) | 7 (36.8) | 5 (83.3) |
| Hyperlipidaemia |  |  |  |
| *No [%]* | 42 (59.2) | 11 (57.9) | 5 (83.3) |
| *Yes [%]* | 29 (40.8) | 8 (42.1) | 1 (16.7) |
| Obstructive sleep apnoea |  |  |  |
| *No [%]* | 50 (70.4) | 14 (73.7) | 4 (66.7) |
| *Yes [%]* | 21 (29.6) | 5 (26.3) | 2 (33.3) |
| Incretin-based therapy use |  |  |  |
| *None [%]* | 34 (47.9) | 12 (63.2) | 4 (66.7) |
| *Semaglutide [%]* | 13 (18.3) | 5 (26.3) | 2 (33.3) |
| *Tirzepatide [%]* | 24 (33.8) | 2 (10.5) | 0 (0.0) |

Data are presented as mean ± standard deviation, median (interquartile range), or number [column percentage] where appropriate. *indicates n = 66 for low risk and n = 17 for indeterminate risk. ALT, alanine aminotransferase; ASCVD, atherosclerotic cardiovascular disease; BMI, body mass index; CAP, controlled attenuation parameter; CKD, chronic kidney disease; HbA1c, glycated haemoglobin; IMD, index of multiple deprivation; LSM, liver stiffness measurement; VCTE, vibration-controlled transient elastography.
