# Supplementary material for: Vibration‐Controlled Transient Elastography (VCTE)‐Based Prevalence and Assessment of Hepatic Steatosis and Fibrosis in a UK Tertiary Hospital‐Based Specialist Weight Management Service: A Real‐World Evaluation
Source: Obes Sci Pract. 2026 Nov 5;12(6):e70204. doi: 10.1002/osp4.70204 (PMC13735886; doi:10.1002/osp4.70204)
Supplement: Supplementary file 3 [file OSP4-12-e70204-s003.docx]

**Supplementary Table 4.** Sensitivity analysis of the multivariable models of factors associated with VCTE-assessed hepatic steatosis (CAP) and fibrosis (LSM) with additional adjustment for incretin-based therapy use.

| **Variable** | **CAP** | | | **LSM** | | |
| --- | --- | --- | --- | --- | --- | --- |
|  | **ß-coefficient** | **95% CI** | ***P*-value** | **ß-coefficient** | **95% CI** | ***P*-value** |
| Age (per year) | -0.13 | -0.89 to 0.64 | 0.745 | -0.01 | -0.04 to 0.03 | 0.723 |
| Female sex | -1.27 | -27.60 to 25.07 | 0.925 | 0.21 | -1.21 to 1.63 | 0.772 |
| Non-white ethnicity | 10.21 | -14.79 to 35.21 | 0.423 | 0.54 | -0.76 to 1.84 | 0.417 |
| IMD (per decile) | 2.25 | -1.02 to 5.53 | 0.178 | 0.05 | -0.12 to 0.21 | 0.572 |
| Alcohol use | -6.89 | -50.20 to 36.43 | 0.755 | 0.94 | -1.56 to 3.43 | 0.462 |
| BMI (per kg/m^2^) | 3.20 | 2.01 to 4.38 | <0.001 | 0.16 | 0.08 to 0.24 | <0.001 |
| HbA1c (per mmol/mol) | 1.35 | 0.15 to 2.55 | 0.027 | 0.13 | 0.05 to 0.21 | 0.002 |
| ALT (per U/L) | 0.68 | 0.03 to 1.33 | 0.039 | 0.04 | 0.00 to 0.08 | 0.050 |
| Incretin-based therapy use | -2.48 | -21.62 to 16.66 | 0.800 | 0.61 | -0.44 to 1.66 | 0.462 |

Data are presented as ß-coefficients, 95% CIs, and *P*-values from multivariable generalised linear models assuming a normal (CAP) or gamma (LSM) distribution with identity link function. ß-coefficients represent the mean change in CAP (dB∙m^-1^) or LSM (kPa) per unit increase in the predictor variable. Binary variables are compared against the reference category (male sex, White ethnicity, no alcohol use, no incretin-based therapy use). Model covariates included age, sex, ethnicity, IMD, alcohol use, BMI, HbA1c, ALT, and incretin-based therapy use. ALT, alanine aminotransferase; BMI, body mass index; CAP, controlled attenuation parameter; CI, confidence interval; HbA1c, glycated haemoglobin; IMD, index of multiple deprivation; LSM, liver stiffness measurement; VCTE, vibration-controlled transient elastography.
