# Supplementary material for: Vibration‐Controlled Transient Elastography (VCTE)‐Based Prevalence and Assessment of Hepatic Steatosis and Fibrosis in a UK Tertiary Hospital‐Based Specialist Weight Management Service: A Real‐World Evaluation
Source: Obes Sci Pract. 2026 Nov 5;12(6):e70204. doi: 10.1002/osp4.70204 (PMC13735886; doi:10.1002/osp4.70204)
Supplement: Supplementary file 4 [file OSP4-12-e70204-s004.docx]

**Supplementary Table 3.** Cohort characteristics stratified by incretin-based therapy use.

| **Characteristic** | **No incretin-based therapy use**  **(*n* = 50)** | **Incretin-based therapy use**  **(*n* = 46)** |
| --- | --- | --- |
| **Demographics** | |  |
| Age (years) | 48.0 ± 12.9 | 49.4 ± 12.1 |
| Sex |  |  |
| *Female [%]* | 41 (82.0) | 39 (84.8) |
| *Male [%]* | 9 (18.0) | 7 (15.2) |
| Ethnicity |  |  |
| *White European [%]* | 41 (82.0) | 37 (80.4) |
| *South Asian [%]* | 8 (16.0) | 7 (15.2) |
| *Black [%]* | 1 (2.0) | 1 (2.2) |
| *Other [%]* | 0 (0.0) | 1 (2.2) |
| IMD (decile) | 7 (5) | 6 (5) |
| Smoking status |  |  |
| *Non-smoker [%]* | 44 (88.0) | 35 (76.1) |
| *Former smoker [%]* | 5 (10.0) | 9 (19.6) |
| *Current smoker [%]* | 1 (2.0) | 2 (4.3) |
| Alcohol use [%] |  |  |
| No *[%]* | 46 (92.0) | 45 (97.8) |
| Yes *[%]* | 4 (8.0) | 2 (2.2) |
| **Anthropometric and biochemical profile** | |  |
| Weight (kg) | 123.7 (25.5) | 104.5 (32.9) |
| BMI (kg/m^2^) | 44.0 (10.0) | 40.6 (10.3) |
| HbA1c (mmol/mol) | 38.0 (6.0) | 37.0 (7.0) |
| ALT (U/L) | 24.5 (16.0) | 20.0 (10.0) |
| Platelet count (×10^9^∙L^-1^) | 274 (66) | 305 (78) |
| **Liver VCTE assessment** | |  |
| CAP (dB/m) | 301 ± 55 | 285 ± 54 |
| Steatosis grade |  |  |
| *S0 (<248 dB∙m^-1^) [%]* | 7 (14.0) | 10 (21.7) |
| *S1 (248-267 dB∙m^-1^) [%]* | 5 (10.0) | 10 (21.7) |
| *S2 (268-279 dB∙m^-1^) [%]* | 5 (10.0) | 4 (8.7) |
| *S3 (≥280 dB∙m^-1^) [%]* | 33 (66.0) | 22 (47.8) |
| LSM (kPa) | 6.3 (3.8) | 5.5 (3.0) |
| Advanced fibrosis risk |  |  |
| *Low (<8 kPa) [%]* | 34 [68.0] | 37 [80.4] |
| *Indeterminate (8-12 kPa) [%]* | 12 [24.0] | 7 [15.2] |
| *High (>12 kPa) [%]* | 4 [8.0] | 2 [4.3] |
| **Medical history** | |  |
| Type 2 diabetes |  |  |
| *No [%]* | 43 (86.0) | 33 (71.7) |
| *Yes [%]* | 7 (14.0) | 13 (28.3) |
| Hypertension |  |  |
| *No [%]* | 27 (54.0) | 25 (54.3) |
| *Yes [%]* | 23 (46.0) | 21 (45.7) |
| Hyperlipidaemia |  |  |
| *No [%]* | 32 (64.0) | 26 (56.5) |
| *Yes [%]* | 18 (36.0) | 20 (43.5) |
| Obstructive sleep apnoea |  |  |
| *No [%]* | 34 (68.0) | 34 (73.9) |
| *Yes [%]* | 16 (32.0) | 12 (26.1) |
| Incretin-based therapy use |  |  |
| *None [%]* | 50 (100.0) | 0 (0.0) |
| *Semaglutide [%]* | 0 (0.0) | 20 (43.5) |
| *Tirzepatide [%]* | 0 (0.0) | 26 (56.5) |

Data are presented as mean ± standard deviation, median (interquartile range), or number [column percentage] where appropriate. *indicates *n* = 47 for the no incretin-based therapy use group and *n* = 42 for the incretin-based therapy use group. ALT, alanine aminotransferase; ASCVD, atherosclerotic cardiovascular disease; BMI, body mass index; CAP, controlled attenuation parameter; CKD, chronic kidney disease; HbA1c, glycated haemoglobin; IMD, index of multiple deprivation; LSM, liver stiffness measurement; VCTE, vibration-controlled transient elastography.
